# Genotype-phenotype associations in *CRB1* bi-allelic patients: a novel mutation, a systematic review and meta-analysis

**DOI:** 10.1186/s12886-024-03419-4

**Published:** 2024-04-15

**Authors:** Ahmad Daher, Malak Banjak, Jinane Noureldine, Joseph Nehme, Said El Shamieh

**Affiliations:** 1 Medical Testing Laboratory, Department of Medical Laboratory Technology, Faculty of Health Sciences, Beirut Arab University, Beirut, Lebanon; 2 Rammal Hassan Rammal Research Laboratory, PhyToxE Research Group, Faculty of Sciences, Lebanese University, Nabatieh, Lebanon; 3 Faculty of Medicine, Holy Spirit University of Kaslik (USEK), Jounieh, Lebanon

**Keywords:** *CRB1*, Rod-cone dystrophy, Leber congenital Amaurosis, Genotype-phenotype

## Abstract

**Purpose:**

The goal of the study was to search for novel bi-allelic *CRB1* mutations, and then to analyze the *CRB1* literature at the genotypic and phenotypic levels.

**Approach:**

We screened various variables such as the *CRB1* mutation types, domains, exons, and genotypes and their relation with specific ocular phenotypes. An emphasis was given to the bi-allelic missense and nonsense mutations because of their high prevalence compared to other mutation types. Finally, we quantified the effect of various non-modifiable factors over the best-corrected visual acuity oculus uterque (BCVA OU) using multivariate linear regression models and identified genetic interactions.

**Results:**

A novel bi-allelic missense in the exon 9 of *CRB1*; c.2936G > A; p.(Gly979Asp) was found to be associated with rod-cone dystrophy (RCD). *CRB1* mutation type, exons, domains, and genotype distribution varied significantly according to fundus characteristics, such as peripheral pigmentation and condition, optic disc, vessels, macular condition, and pigmentation (*P* < 0.05). Of the 154 articles retrieved from PubMed, 96 studies with 439 bi-allelic *CRB1* patients were included. Missense mutations were significantly associated with an absence of macular pigments, pale optic disc, and periphery pigmentation, resulting in a higher risk of RCD (*P* < 0.05). In contrast, homozygous nonsense mutations were associated with macular pigments, periphery pigments, and a high risk of LCA (*P* < 0.05) and increased BCVA OU levels. We found that age, mutation types, and inherited retinal diseases were critical determinants of BCVA OU as they significantly increased it by 33% 26%, and 38%, respectively (*P* < 0.05). Loss of function alleles additively increased the risk of LCA, with nonsense having a more profound effect than indels. Finally, our analysis showed that p.(Cys948Tyr) and p.(Lys801Ter) and p.(Lys801Ter); p.(Cys896Ter) might interact to modify BCVA OU levels.

**Conclusion:**

This meta-analysis updated the literature and identified genotype-phenotype associations in bi-allelic *CRB1* patients.

**Supplementary Information:**

The online version contains supplementary material available at 10.1186/s12886-024-03419-4.

## Introduction

Biallelic mutations of the *CRB1 (crumbs cell polarity complex component 1)* gene (MIM: 604,210) are a rare cause of inherited retinal dystrophies (IRDs) [1]. Altogether, they have been associated with several clinical features ranging from Leber congenital amaurosis (LCA) to rod cone dystrophy (RCD), with LCA being the most severe case of progressive retinal degeneration [2]. This difference in disease severity may be attributed to LCA being due to defective retinal progenitor cells during early ocular development, while RCD is caused by faulty photoreceptor-muller cell interaction [3]. Previous reports show that 9 to 15% of LCA cases [4] and 6.5% [5] of RCD cases result from biallelic *CRB1* mutations.

*CRB1*-associated retinopathy has specific features, such as the early onset of maculopathy, the loss of retinal lamination associated with increased retinal thickness, nummular intraretinal pigmentation [6], the preservation of the para-arteriolar retinal pigment epithelium, optic drusen [7] and the existence of macular cysts [8]. Despite that, the expression of the retinal phenotype can vary, even within families, and several genetic or environmental factors contribute [9].

*CRB1* encodes a protein consisting of 12 exons which are subject to alternative splicing at its 3’ end to produce two isoforms of 1376 and 1406 amino acids, respectively [1]. Both isoforms comprise 19 epidermal growth factor (EGF)-like domains, three laminin A globular (AG)-like domains, and a signal peptide sequence, with the longer isoform containing an additional transmembrane and cytoplasmic domain [1, 10]; with the latter being formed of the conserved FERM and PDZ motifs that connect the CRB1 protein to the actin cytoskeleton [10, 11]. At the genotypic level, the c.2843G > A; p.(Cys948Tyr) mutation is the most prevalent with 12.48%, followed by c.2401 A > T; p.(Lys801Ter) with 3.5% and c.2234 C > T; p.(Thr745Met) with 3.45% of all reported alleles [12].

Despite many studies, no clear correlation between *CRB1* genotypes and related ocular phenotypes has been established [8, 9]. Identifying such relationships might pave the way for genetic counseling and developing targeted therapies [13, 14]. We have shown the presence of mutational hotspots in *CRB1* at exons 2, 6, and 7 [22]. Specifically, the indels and nonsense mutations in EGF like and Laminin G like domains of exons 2, 6, and 7 were associated with non-arRCD (mainly LCA and EORD) [22]. To go further, herein we first reported a novel bi-allelic *CRB1* mutation associated with RCD and then analyzed the *CRB1* literature at the genotypic and phenotypic levels from 439 patients worldwide—all of the above to identify specific *CRB1* genotype-phenotype associations.

## Methods

### Ethics statement

All our procedures were conducted according to the principles outlined in the Declaration of Helsinki. The Institutional Review Board of Beirut Arab University approved our study (2017 H-0030-HS-R-0208). The participants provided written informed consent; their ophthalmic examinations were done at the Beirut Eye and ENT Specialist Hospital (Beirut, Lebanon).

### Whole-exome sequencing, pathogenicity interpretation, and co-segregation analysis

Family 23 is part of a larger cohort of Lebanese individuals with different IRD conditions collected since 2015, it was included because it reports a bi-allelic *CRB1* mutation.DNA extraction using the QIAamp DNA Mini Kit (Hilden, Germany) from Qiagen. Whole exome sequencing (WES) was conducted as described previously [15]. All the exons (∼ 22,000 genes) were captured by xGen Exome Research Panel v2 (Integrated DNA Technologies, Coralville, Iowa, USA). The captured regions of the genome were sequenced with Novaseq 6000 (Illumina, San Diego, CA, USA). The raw genome sequencing data analysis, including alignment to the GRCh37/hg19 human reference genome, variant calling, and annotation, was conducted with open-source bioinformatics tools and in-house software developed by 3 Billion company (Seoul, Korea). Unidirectional Sanger sequencing was applied to all available family members’ DNA to analyze the co-segregation.

### Literature search, study selection, and data collection

Our systematic review analyzed the data from published articles on *CRB1* bi-allelic mutations containing ocular data. Our protocol was based on the preferred reporting items for systematic reviews and meta-analyses (PRISMA) [16]. The review was not registered. All the identified *CRB1* variants reported on The Human Gene Mutation Database (HGMD) [17] (last accessed on December 2021) were downloaded. Then, each variant was searched on Clinvar (NCBI) (last accessed on December 2022) to obtain all their corresponding articles. We also performed a manual Medline search that retrieved articles published until February 2023. The most critical inclusion criterion was clinical and genotypic data availability. In contrast, the exclusion criteria were: (1) the absence of ocular data. (2) The use of non-English language (because of the language barrier). (3) The absence of full text. The included articles were distributed amongst four authors to collect patients’ clinical and genotypic data and were further homogenized. The previous filtering steps led to 96 articles published since 1999. The PRISMA flow diagram was summarized in Supplementary data.

### Ocular and clinical data

The ophthalmologist analyzed the fundus autofluorescence (FAF) and optical coherence tomography images and further classified them. FAF variables included; (1) macular condition classified into; normal, yellow macular degeneration (YMD), schizis, no reflex, edema, deposits, degeneration, coloboma like, Bull’s maculopathy, and atrophy. (2) Macular pigmentation classified into; pigmentation presence (yes/no), nummular, granular, bone spicule, and beaten metal), optic disc state (normal, pseudo papilledema, pale, hyperemic, granular, gliotic, and drusen), vessels’ state (normal, tortuosity, preserved para arteriole (retinal pigment epithelium) RPE, no perivascular sheathing, narrowed, edema, constricted, attenuated, para arteriolar RPE changes, and perivascular pigmentation). (3) Peripheral condition; normal, salt and pepper, RPE changes, RPE atrophy, loss of RPE, deposits, degenerative fundus, and atrophy.

(4) Peripheral pigmentation; normal, marbleized, bone spicule, salt and pepper, and unspecified pigmentation. Optical coherence tomography (OCT) images description were classified into normal, no macular atrophy, cystoid macular edema (CME), no CME, macular thinning, macular thickening, macular atrophy, hyperreflective, and degeneration.

### Statistical analysis

All analyses were conducted using SPSS software version 26 (SPSS, Inc, Chicago, Illinois). Clustered bar charts and Boxplots were generated using Origin software (OriginPro, Version 2022, OriginLab Corporation, Northampton, MA, USA). For statistical power reasons, variables with a sample size of less than five were omitted. Categorical variables such as the type of IRD, genotype, and clinical data were expressed as percentages. Continuous variables such as age, BCVA OU, and refraction were expressed as mean ± standard deviation. BCVA OU data were obtained as Snellen fractions and then transformed into LogMAR scale. BCVA OU was also transformed into a binary variable based on its median (1.3 LogMar). A chi-square (χ2) test of independence was used to test the difference in proportion between the categorical variables, and the ocular, and clinical data. Kruskal-Wallis one-way ANOVA and Mann-Whitney U tests were used to compare the BCVA OU according to several categorical variables. For the multiple linear regression models over BCVA OU levels, we used the following as independent variables; age, gender, mutation type, and IRD (the three most prevalent disorders; RCD, LCA, and retinal dystrophy).

The significance level was set at *P* ≤ 0.05.

## Results

**Fig. 1:**
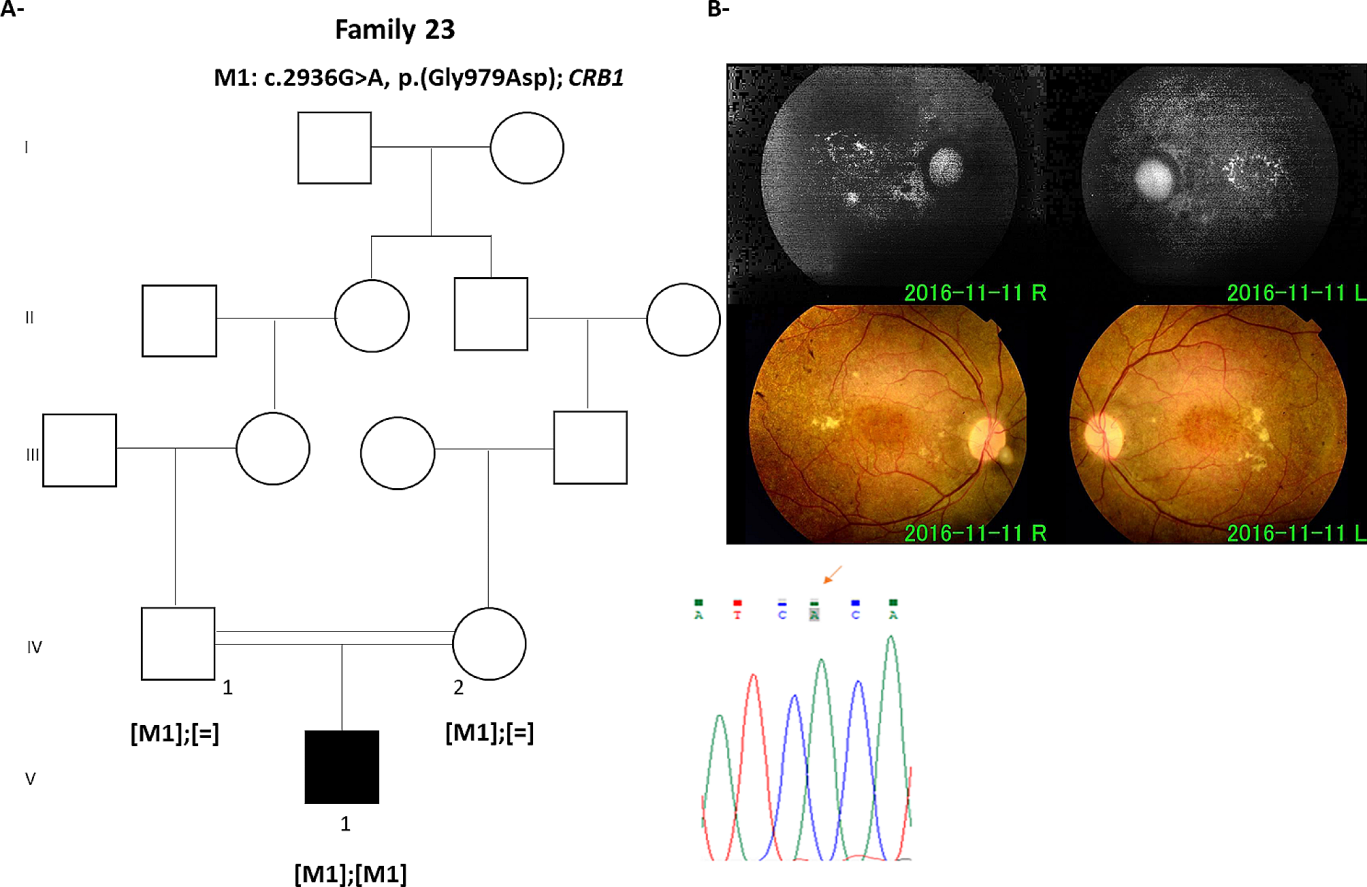
The novel missense bi-allelic mutation c.2936G > A; p.(Gly979Asp) in crumbs cell polarity complex component 1 *CRB1* **A**- Pedigree of the family with an index patient having a homozygous c.2936G > A; p.(Gly979Asp) in *CRB1*. **B**- Fundus auto-fluorescence images of the index patient (F23:V.1). Black symbol indicate affected member. Square and round symbols represent male and female individuals, respectively. The slash indicates deceased individuals. [M] defines mutated alleles. [ =] defines wild type alleles

In family 23, index F23:V.I is a 23 years-old male diagnosed with RCD at age 16. His parents are second cousins (Fig. 1A). Colour fundus photographs revealed macular degeneration with peripheral deposits and pale optic disk (Fig. 1B). His ERG exhibited a very reduced photopic and scotopic responses. F23:V.1 carries a homozygous missense mutation; (NM_201253.3): c.2936G > A; p.(Gly979Asp) in exon 9 (GRCH38: g.197434799G > A). This mutation is a novel missense variant not reported in available population databases such as ExAC, gnomADand HGMD, more importantly, it was not previously associated with RCD. The identified missense affects a conserved residue across species Glycine residue at amino acid position; 979. This variation was predicted to be probably damaging, damaging, and disease causing according to Polyphen, SIFT and mutalyzer tools respectively. The American College of Medical Genetics (ACMG) classification showed it as a PP2 variant. Sanger sequencing analysis validated the presence of this homozygous mutation in the index patient. Both parents carried the mutation (Fig. 1A).

**Fig. 2:**
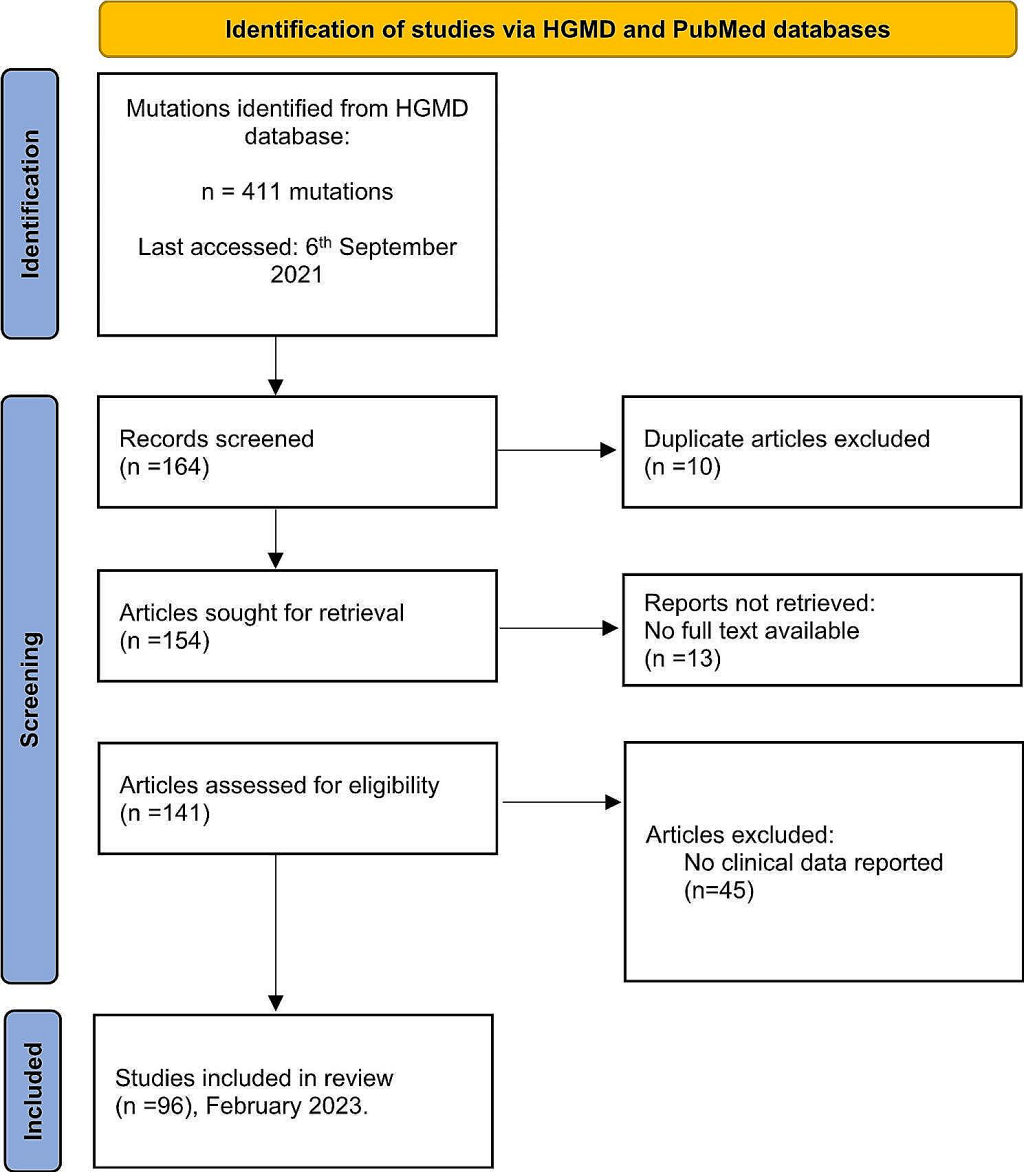
Flow chart for Identification of eligible studies

In addition, we have conducted a systematic review of the known *CRB1* bi-allelic mutations that were found to have clinical data. This led to 96 articles and 439 bi-allelic *CRB1* patients (Fig. 2). The included individuals were young, having an average age of 19 years, with 54% being females (Table 1). The participants were from all over the world, with the majority (∼ 91%) from Asia (56%) and Europe (35%) (Table 1).

**Table 1:** Demographic characteristics of the affected individuals with bi-allelic *crumbs cell polarity complex component 1* (*CRB1*) mutations

Demographic characteristics	Participants (*N* = 439)	
	Mean^a^	SD^b^
**Age at baseline (y) (*n*** = 345)	19.1	15.2
**Age of Onset (y) (** ***n*** ** = 218)**	6.4	8.2
**Gender (female %)**	169 (54%)	
**Continent(** ***n*** ** = 318)**		
Asia	180 (56%)	
Europe	108 (35%)	
South America	19 (6%)	
North America	6 (1%)	
Australia	3 (1%)	
Africa	2 (1%)	
**Country (** ***n*** ** = 318)**		
China	113 (35%)	
Belgium	23 (7%)	
Brazil	17 (5%)	
Netherlands	14 (4%)	
Spain	14 (4%)	
Israel/Palestine	13 (4%)	
France	11 (2%)	

Values are arithmetic mean ± SD for continuous variables. Categorical variables are shown as numbers (n) and percentages (%).In the country analysis, a frequency threshold of 2% was used; thus, countries with frequencies ≤ 1% were not shown. n: sample size

The clinical characteristics of all included patients were summarized in Table 2. 43% of the included patients had LCA, 37% had RCD, and 9% did not receive a detailed diagnosis (classified as retinal degeneration). The average BCVA OU was 1.6 LogMAR units. Fundus images revealed that the majority of patients showed Macular atrophy (*n* = 60, 32%), with no changes in macular pigmentations (*n* = 114, 48%), pale optic disc (*n* = 37, 51%), attenuated vessels (*n* = 28, 29%), unspecified pigmentation (*n* = 102, 68%) accompanied by fundus degeneration (*n* = 30, 64%) in the periphery and Cystoid Macular Edema (*n* = 32,40%) in OCT. Regarding symptoms, most had night blindness, nystagmus, and poor vision (Table 2).

**Table 2:** Clinical characteristics of the affected individuals with bi-allelic *crumbs cell polarity complex component 1* (*CRB1*) mutations

Clinical characteristics	Participants (*N* = 439)	
	Mean^a^	SD^b^
**IRD condition (** ***n*** = 372)*		
LCA	159 (43%)	
RCD	125 (37%)	
RD	32 (9%)	
Early Onset Retinal Dystrophy	30 (7%)	
Other	33 (9%)	
**Vision**		
BCVA OU (LogMAR, *n* = 331)	1.6	1.1
Refraction OU Spherical (*n* = 54)f	5.6	2.8
**Fundus Characteristics**		
**Macular condition (** ***n*** ** = 188)**		
Atrophy	60 (32%)	
Yellow Macular Degeneration	37 (20%)	
Degeneration	30 (16%)	
Deposits	27 (14%)	
Others	34 (18%)	
**Macular Pigmentation (** ***n*** ** = 241)**		
No	114 (48%)	
Nummular	62 (28%)	
Bone Spicule	37 (15%)	
Other	28 (9%)	
**Optic Disc (** ***n*** ** = 72)**		
Pale	37 (51%)	
Normal	22 (31%)	
Drusen	7 (10%)	
Others	6 (8%)	
**Vessels (** ***n*** ** = 96)**		
Attenuated	28 (29%)	
Preserved para arteriole RPE	22 (22%)	
Para arteriolar RPE changes	18 (19%)	
Narrowed	14 (15%)	
Others	14 (15%)	
**Peripheral pigmentation (** ***n*** ** = 149)**		
Pigmentation	102 (68%)	
Bone Spicule	32 (21%)	
Salt and pepper	12 (8%)	
Others	3 (3%)	
**Peripheral condition (** ***n*** ** = 47)**		
Degenerative fundus	30 (64%)	
Loss of RPE	4 (8%)	
RPE atrophy	3 (6%)	
Others	10 (22%)	
**Optical Coherent Tomography**		
**Macular Thickness (** ***n*** ** = 79)**		
CME	32 (40%)	
no CME	16 (20%)	
Macular Thickening	12 (15%)	
Others	19 (25%)	
**Symptoms**		
**Cataract (** ***n*** ** = 25)**		
Yes N (%)	17 (68%)	
**Night Blindness (** ***n*** ** = 101)**		
Yes N (%)	98 (97%)	
**Nystagmus (** ***n*** ** = 219)**		
Yes N (%)	177 (81%)	
**Keratoconus (** ***n*** ** = 18)**		
Yes N (%)	17 (94%)	
**Strabismus (** ***n*** ** = 14)**		
Yes N (%)	12 (86%)	
**Photophobia (** ***n*** ** = 25)**		
Yes N (%)	23 (92%)	
**Poor Vision (** ***n*** ** = 142)**		
Yes N (%)	138 (97%)	
**Constricted Visual Field (** ***n*** ** = 14)**		
Yes N (%)	14 (100%)	

Values are arithmetic mean ± SD for continuous variables. Categorical variables are shown as numbers (n) and percentages (%). n: sample size

* *N* = 372, as 69 *CRB1* bi-allelic patients did not receive a definitive diagnosis

IRD: inherited retinal disease, LCA: Leber congenital amaurosis, RCD: Rod Cone Dystrophy, RD: Retinal Dystrophy. BCVA: Best Corrected Visual Acuity, OU: Oculus Uterque (both eyes)

The most prevalent mutations were the bi-allelic; p.(Cys948Tyr) (*n* = 15,3%), p.(Gly1103Arg) (*n* = 13, 3%) and p.(Gly614Val) (*n* = 8, 2%) (Table 3). In addition to that, most biallelic mutations were in both Laminin G-like 2 domains (*n* = 51, 12%), Laminin G-like 1 (*n* = 44, 10%) and EGF-like 14 (*n* = 24, 6%, Table 3). Moreover, approximately half of the bi-allelic *CRB1* were homozygous missense mutations (51%, Table 3), followed by the compound heterozygous missense, indels (12%, Table 3), and missense and nonsense (11%, Table 3).

**Table 3:** Genetic characteristics of the affected individuals with bi-allelic *crumbs cell polarity complex component 1* (*CRB1*) mutations

Genetic characteristics	Participants (*N* = 439)
	*N* (%)
**Exons combinations (** ***n*** ** = 438)**	
6 and 6	69 (16%)
7 and 7	45 (10%)
9 and 9	34 (8%)
8 and 8	23 (5%)
7 and 9	21 (5%)
6 and 7	19 (4%)
6 and 9	15 (3%)
**Domains combinations (** ***n*** ** = 430)**	
laminin G-like 2 and laminin G-like 2	51 (12%)
Laminin G-like 1 and Laminin G-like 1	44 (10%)
EGF-like 14 and EGF-like 14	24 (6%)
Laminin G-like 3 and Laminin G-like 3	21 (5%)
Laminin G-like 2 and EGF-like 14	15 (3%)
EGF-like 9 and EGF-like 9	13 (3%)
EGF-like 17 and EGF-like 17	9 (2%)
**Genotype (** ***n*** ** = 439)**	
Homozygous p.(Cys948Tyr)	15 (3%)
Homozygous p.(Gly1103Arg)	13 (3%)
Homozygous p.(Gly614Val)	8 (2%)
p.(Cys948Tyr) and c.2978 + 5G > A	7 (2%)
p.(Lys801Ter) and p.(Cys896Ter)	7 (2%)
Homozygous p.(Cys383Tyr)	6 (1%)
p.(Leu1074Ser) and c.2677–2 A > C, p.=	6 (1%)
**Mutations types (** ***n*** ** = 439)**	
Missense and Missense	222 (51%)
Missense and Indel	53 (12%)
Missense and Nonsense	49 (11%)
Nonsense and Nonsense	36 (8%)
Indel and Indel	28 (6%)
Missense and Splice site	25 (6%)
Splice site and Splice site	11 (2%)

Categorical variables are shown as numbers (n) and percentages (%). n: sample size

When investigating a possible association between *CRB1* genotypes and the ocular phenotypes using the χ2 independent test (Table 4), we found that mutation types differed significantly according to fundus characteristics; peripheral pigmentation (*P* = 0.002, Table 4) and peripheral condition (*P* = 0.009, Table 4), optic disc (*P* = 0.02, Table 4), macular pigmentation (*P* < 0.0001, Table 4) and condition (*P* = 0.002, Table 4). CRB1 domains also differed according to peripheral pigmentation (*P* = 0.022, Table 4) and peripheral retina condition (0.004, Table 4), state of optic vessels (*P* = 0.001, Table 4), macular pigmentation (*P* = 0.025, Table 4) and macular condition (*P* < 0.0001, Table 4). As for the exons, there was a statistically significant association with the optic vessels (*P* = 0.003, Table 4) and disc (*P* = 0.006, Table 4) and macular condition (*P* = 0.006, Table 4). *CRB1* genotypes varied significantly in peripheral pigmentation (*P* = 0.015, Table 4), macular pigmentation (*P* = 0.008, Table 4), and macular condition (*P* < 0.0001, Table 4).

**Table 4:** Genetic associations of *crumbs cell polarity complex component 1* (*CRB1) *mutation types, domains, exons and genotypes with the the fundus autofluorescence characteristics

Fundus characteristics	CRB1			
	Mutations Types	Domain	Exons	Genotype
Peripheral Pigmentation	0.002	0.022	N. S	0.015
Peripheral retina condition	0.009	0.004	N. S	N. S
Optic Vessels	N. S	0.001	0.036	N. S
Optic Disc	0.02	N. S	0.006	N. S
Macular Pigmentation	< 0.0001	0.025	0.006	0.008
Macular Condition	0.002	< 0.0001	N. S	< 0.0001

A χ2 test of independence was used to test the association between *CRB1* variables and the fundus autofluorescence characteristics

The associations with the refraction and OCT macular thickness were not shown since it didn’t reach the significance level

N.S: not significant

To go further, we focused on the mutation types and their relation with specific ocular phenotypes since they were the most abundant using the χ2 independent test. Our analysis showed that the presence of homozygous missense mutations was significantly associated with an absence of macular pigments, pale optic disc, and presence of periphery pigmentation that resulted in a 1.5x increased risk of RCD compared to LCA (60% vs. 40% respectively, Table 5). The presence of a missense and nonsense was significantly associated presence of periphery pigments and a higher risk of LCA (63% vs. 40% respectively Table 5). When the nonsense replaced an indel, the latter was significantly associated with macular atrophy, no macular pigmentation, a pale optic disc, and a higher risk of LCA (*P* = 72% vs. 63% respectively, Table 5). The homozygous nonsense in *CRB1* was significantly associated with macular pigments, periphery pigments, and a high risk of LCA (90%, Table 5). The BCVA OU levels increased proportionally (almost 2x) when comparing individuals carrying homozygous missense mutation to those having null mutations (1.4 vs. 2.7, *P* = 0.001, Table 5). This shows that the addition of null alleles additively increases the risk of LCA and thus increasing the BCVA OU values proportionally with the nonsense having a more profound effect compared to indels.

**Table 5:** Association of crumbs cell polarity complex component 1 (*CRB1)* mutation types with retinal and clinical characteristics

CRB1	Fundus characteristics				BCVA OU	IRDs	
Mutation types	Macular Condition (Xi^2^ = 65, *P* = 0.026)	Macular pigmentation (Xi^2^ = 44, *P* = 0.01)	Optic Disc (Xi^2^ = 63, *P* < 0.001)	Periphery pigmentation (Xi^2^ = 33, *P* = 0.034))	(LogMar, *P* = 0.001)	(Xi^2^ = 41, *P* = 0.001)	
	Atrophy	Absence	Pale	Presence	Relative risk	RCD	LCA
Homozygous Missense		56 (49%)	18 (46%)	38 (63%)	1.4	88 (60%)	59 (40%)
Missense; Nonsense				15 (79%)	1.7	12 (37%)	20 (63%)
Missense; Indel	13 (65%)	12 (43%)	6 (67%)		1.5	9 (28%)	23 (72%)
Homozygous Nonsense		14 (50%)		14 (78%)	2.7	3 (10%)	28 (90%)

The relative risk was calculated using a logistic binary model with BCVA OU as the dependent variable and the CRB1 mutation types as independent

BCVA: Best Corrected Visual Acuity, OU: Oculus Uterque, IRDs: inherited retinal diseases, Xi^2^: Chi-square independent test, RCD: Rod Cone Dystrophy, H: H Kruskal Wallis, LCA: Leber congenital amaurosis

Multivariate linear regression models were used to test the relative effects of various independent variables such as age, gender, mutation type, genotype, and IRD condition on the BCVA OU (Table 6). The four models explained between 8% and 15% of the BCVA OU variability (*P* = 0.001, Table 6). In model 3 explaining 14% of the BCVA OU variance, we found that age and mutation types were associated with 33% and 26%, respectively, increase in BCVA OU that is correlated with a decreased vision (Table 6). In model 4, age and IRD condition were associated with a 25% and 38% respective increase in BCVA OU (Table 6). This indicates that mutation types and IRD conditions are key to determining visual acuity. In contrast, gender and genotypes showed no significant association (*P* > 0.05. Table 6).

**Table 6:** Association between different demographic, genetic and phenotypic characteristics and the best-corrected visual acuity using multiple linear regression models

Characteristics	Best Corrected Visual Acuity OU (LogMAR)							
	Model 1 (R^2^ = 0.08, *P* = 0.001)		Model 2 (R^2^ = 0.92,*P* = 0.001)		Model 3 (R^2^ = 0.14,*P* = 0.001)		Model 4 (R^2^ = 0.15,*P* = 0.001)	
	β	*P*	β	*P*	β	*P*	β	*P*
**Age**	0.272	< 0.001	0.273	< 0.001	0.332	0.001	0.254	0.003
**Gender**	-0.07	0.24	-0.08	0.221	-0.05	0.269	-0.12	0.152
**Mutation types**					0.26	0.001		
**Genotype**			-0.108	0.09				
IRDs*							0.38	< 0.001

β = standardized linear regression coefficient

BCVA: Best Corrected Visual Acuity, OU: Oculus Uterque, IRDs: inherited retinal diseases

IRDs*: used as binary variable rod-cone dystrophy and Leber congenital amaurosis

**Fig. 3:**
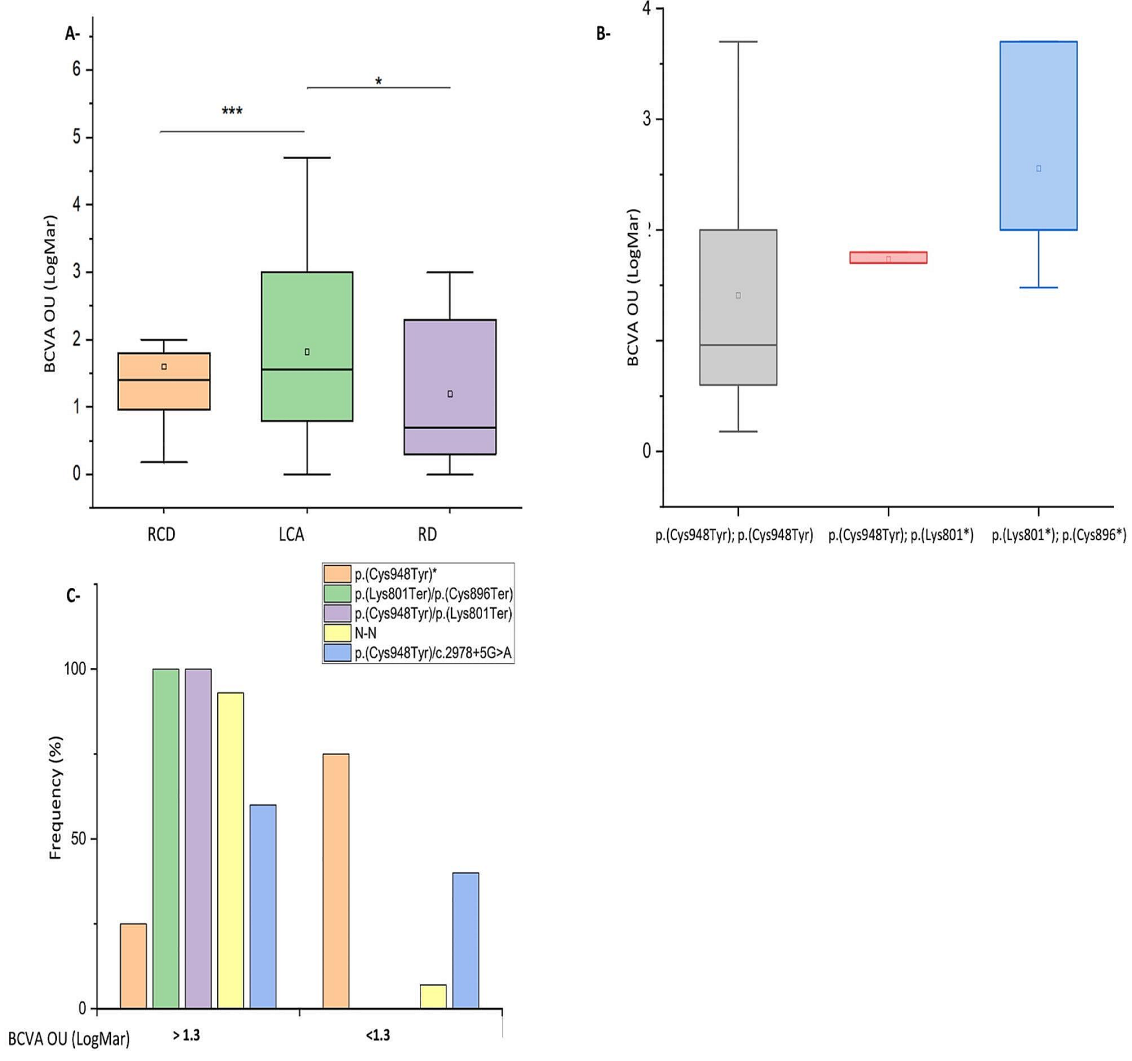
Best corrected visual acuity variation according to phenotype, mutation types, and specific mutations A- Boxplot showing the variation of BCVA OU (Log MAR) with respect to phenotype. B- Boxplot showing the variation of BCVA OU (Log MAR) according to several *CRB1* bi-allelic mutations. C- Distribution of BCVA OU used as a binary variable (threshold 1.3 LogMar) according to several *CRB1* bi-allelic mutations * *P* < 0.05, ***P < 0.001 RCD: Rod-cone dystrophy, RD: Rod Dystrophy, LCA: Leber Congenital Amaurosis, BCVA: Best Corrected Visual Activity, OU: Oculus Uterque

The effect of different variables on BCVA OU were shown in Fig. 3. The highest BCVA OU (inversely correlated with vision) were attributed to LCA (Fig. 3A) and to biallelic nonsense mutations (Fig. 3B). In homozygous p.(Cys948Tyr) patients, BCVA OU was around 1.4 LogMar (Fig. 3B). When one p.(Cys948Tyr) allele was replaced by the nonsense p.(Lys801Ter) the BCVA OU increased by 21% (1.7, Fig. 3B). Similarly, in compound heterozygous patients p.(Cys948Tyr); p.(Cys896Ter) BCVA OU was 2.6 LogMar. When BCVA OU was stratified (according to 1.3 LogMar). Precisely, 75% of p.(Cys948Tyr) individuals had a low BCVA OU, whereas all the compound heterozygous p.(Cys948Tyr); p.(Lys801Ter) and p.(Cys896Tyr); p.(Lys801Ter) had high BCVA OU (Fig. 3C). The above observations point out a possible interaction between p.(Cys948Tyr), and p.(Lys801Ter), and between p.(Lys801Ter), and p.(Cys896Ter) over the BCVA OU in bi-allelic *CRB1* patients.

We have also studied the repartition of the mutated exons across continents and found that Asians harbored most of their bi-allelic *CRB1* mutations in exons 6, 9, and 7. In contrast, Europeans exhibited most of their bi-allelic mutations in exons seven and eight (Supplementary Figure S1). Similarly, despite being the most reported mutation worldwide [12], p.(Cys948Tyr) has not been reported in Asian individuals yet at least in those with ocular data (Supplementary Figure S2). This observation might point out for ethnic specificity in terms of *CRB1* mutational hotspots.

## Discussion

Herein, we first identified a novel bi-allelic missense in the exon 9 of *CRB1*; c.2936G > A; p.(Gly979Asp) associated with RCD. This mutation co-segregated in all the family members. *CRB1* mutations are associated with different IRDs ranging from milder forms of RCD and CRD to more severe forms such LCA and as early onset retinal dystrophy [18, 19]. As in previous studies, our meta-analysis showed that LCA/EORCD is the most prevalent IRD condition, followed by RCD [5]. Analyzing the genotypic and phenotypic data from 439 multi-ethnic bi-allelic *CRB1* patients validated the previous findings concerning its most prevalent mutations; p.(Cys948Tyr), p.(Gly1103Arg), p.(Gly614Val) and exons (7 and 9).

Because of the relatively smaller sample size, many previous studies reported no association between the type of *CRB1* mutation, its location, and the IRD form or its related ocular characteristics. In contrast, our meta-analysis showed that *CRB1* mutation type, exons, domains and genotypes distribution vary significantly according to the fundus characteristics such as the peripheral pigmentation and condition, the optic disc and vessels, the macular condition and pigmentation.

To go further, we focused on the mutation types and their relation with specific ocular phenotypes. We found that bi-allelic missense mutations were significantly associated with an absence of macular pigments, pale optic disc, and presence of periphery pigmentation, which resulted in a 1.5 x increased risk of RCD, compared to LCA. The presence of a missense and nonsense was significantly associated with periphery pigments and a higher risk of LCA. When the nonsense was replaced by an indel, a significant association with macular atrophy, no macular pigmentation, a pale optic disc, and much higher risk of LCA was found. The bi-allelic nonsense mutations were associated with macular pigments, periphery pigments, and a high risk of LCA reaching 90%. The BCVA OU levels increased proportionally when comparing individuals carrying homozygous missense mutation to those having null mutations. The above results reveal specific ocular association and shows that the addition of null alleles additively increases the risk of LCA thus increasing the BCVA OU levels with the nonsense having a more profound effect than indels. This is biologically valid since missense mutation may still lead to a residual protein function and, therefore, would be expected to cause a milder form of the disease, such as RP [8, 20]. In contrast, null alleles have higher loss-of-function effects and are associated with more severe phenotypes (LCA) [8].

When the BCVA OU was compared across different genotypes, homozygous p.(Cys948Tyr) patients had lower values compared to p.(Cys948Tyr); p.(Lys801Ter) (21% less). Similarly, p.(Cys948Tyr); p.(Cys896Ter) patients had even higher BCVA OU. This points out for an interaction between these alleles over the BCVA OU. The stratification of the BCVA OU according to 1.3 LogMAR threshold (Blindness is defined as a BCVA worse than 1.3 LogMAR), replicated the previous results. Den Hollander et al. [21] previously suggested that the homozygous p.(Cys948Tyr) is a null mutation where people bearing this mutation show clinical characteristics more similar to LCA than RCD. Our analysis support this finding since LCA patients having the homozygous p.(Cys948Tyr) were ≈ 4x more than those of RCD (53% vs. 13% respectively). However, when the prevalence of individuals having high BCVA OU status was compared between the homozygous p.(Cys948Tyr) and other bi-allelic nonsense mutations (associated with LCA), it showed opposite repartition (25% vs. 93% respectively). Thus, although homozygous p.(Cys948Tyr) is causing more LCA than RCD, it might have less severe effect.

Previous literature suggested that exons 7 and 9 are predominantly mutated [9, 14]. This study replicated these findings and additionally showed that exon 6 is also a hotspot for several mutations among Asian patients. Hence designing targeted panels that cover exons 6, 7 and 9 for patients with suspected *CRB1* mutations will help solve around 62% of the cases at a significantly lower cost.

We have found that all *CRB1* mutation types are associated with macular and peripheral pigmentations. Unfortunately, the type of pigmentary change was not specified which rendered impossible to identify a specific pigmentary change for each mutation type.

We also quantified the relative effect of age, mutation type and IRD condition over the BCVA OU and found that these three variables are key determinants of BCVA OU as they significantly increase it by 33% 26%, and 38% respectively. The effect of the IRD on BCVA was clearly highlighted in Model 4 where its standardized coefficient was 0.38, 1.4 times higher than that of age (0.272, Model 1) and 1.46 times higher than mutation type (0.26, Model 3). This is not surprising since both LCA and RCD have severe effects on visual acuity. However, the importance of these models lies in the fact that the effect of mutation type and age is approximately equal on the visual acuity. Of note, phenotype and mutation type were not included in the same model since these variables are associated (dependent variables) which will alter the linear regression model’s accuracy. None of the proposed models explained the totality of the BCVA OU, highlighting the effect of environmental, epigenetic, and other modifying factors on BCVA.

The current study has several strengths; (1) it is the largest study conducted on *CRB1* mutations. (2) The first using multivariate linear regression models to show the effect of non-modifiable demographics and genetic variables on BCVA OU. It is the first to highlight specific associations with the fundus characteristics.

In contrast, several limitations can be mentioned; (1) We selected the *CRB1* bi-allelic patients having adequate phenotypic and clinical data for further genotype-phenotype association analysis, thus excluding any individual(s) not characterized at the ocular level, even if the genetic information is complete. This selection bias could change the overall prevalence of some *CRB1* mutations. (2) Our analysis could not confirm clinical characteristics such as the IRD condition or other ocular phenotypes. (3) Many duplicate individuals might be included if named differently across studies by the investigators. (4) No association was explored with OCT because of the relatively low number of individuals with adequate measurements.

This study updates the known *CRB1* mutations and reports a novel one in a Lebanese family with RCD. Based on the available literature, we performed specific genotype-phenotype associations between *CRB1* mutation types, domains, exons, and specific genotypes with their associated IRDs and ocular phenotypes. We investigated the relative relation between several non-modifiable factors (age, mutation type, and IRD condition) with the clinical and the BCVA OU variability in bi-allelic *CRB1* patients. Finally, we identify three alleles that might interact to influence the BCVA OU. Future studies, including diverse multi-ethnic participants, can help uncover additional novel mutations and elucidate the global spectrum of *CRB1* mutations. They will also help understand the progression of *CRB1*-associated retinopathies over time, inform clinical management strategies, and identify windows of opportunity for intervention.

### Electronic supplementary material

Below is the link to the electronic supplementary material.

Supplementary Material 1

## Data Availability

The datasets generated and/or analysed during the current study are available in the DRYAD repository; https://datadryad.org/stash/dataset/doi:10.5061/dryad.pg4f4qrwf.
