# Supplementary material for: Genotype-phenotype associations in CRB1 bi-allelic patients: a novel mutation, a systematic review and meta-analysis
Source: BMC Ophthalmol. 2024 Apr 15;24:167. doi: 10.1186/s12886-024-03419-4 (PMC11017593; doi:10.1186/s12886-024-03419-4)
Supplement: Supplementary file 1 — Supplementary Material 1 [file 12886_2024_3419_MOESM1_ESM.docx]

Figure S1: *CRB1* exons and mutations distribution according to continents.


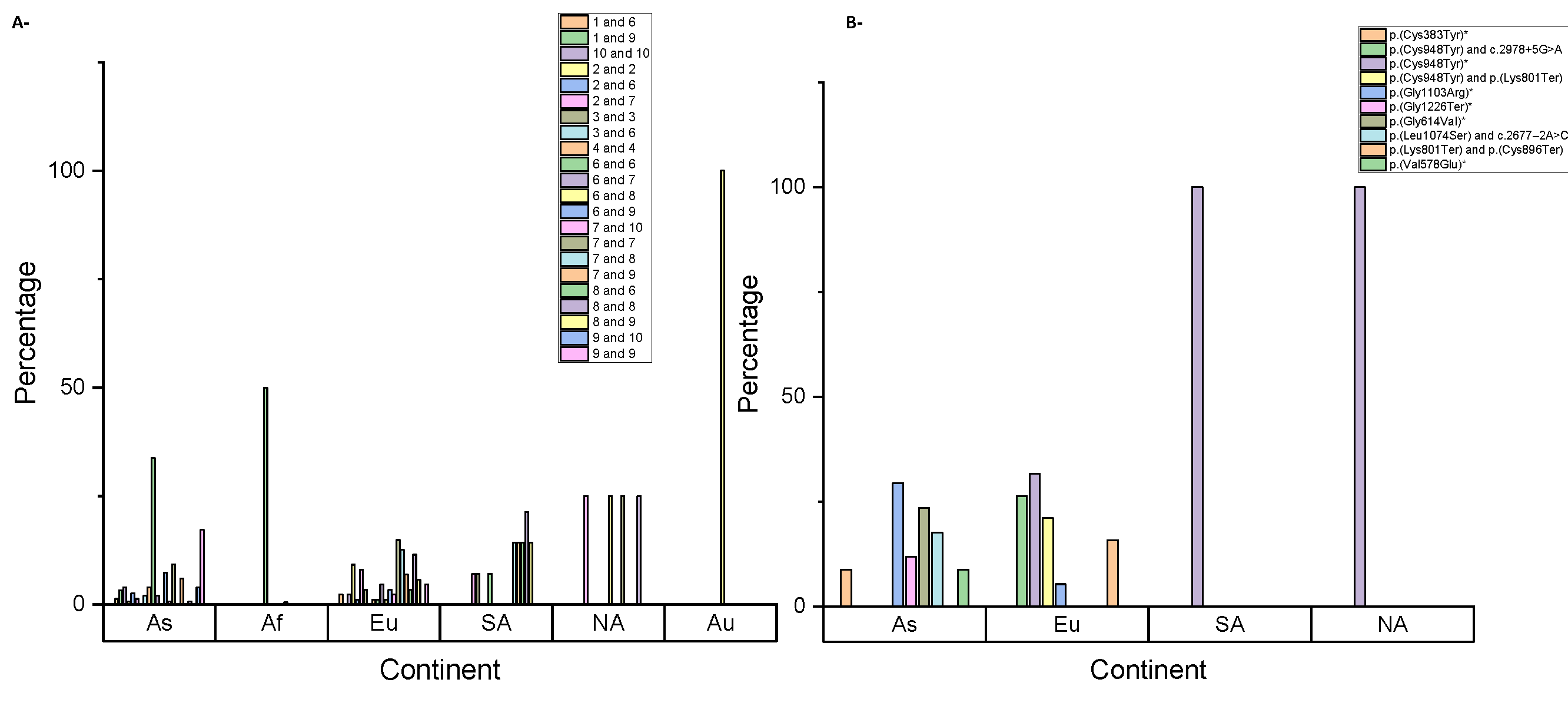


As: Asians, Af: Africans, Eu: Europeans, SA: South Americans, NA: North Americans, Au: Australia.

**Figure S2:** *CRB1* exons and mutations distribution according to continents.


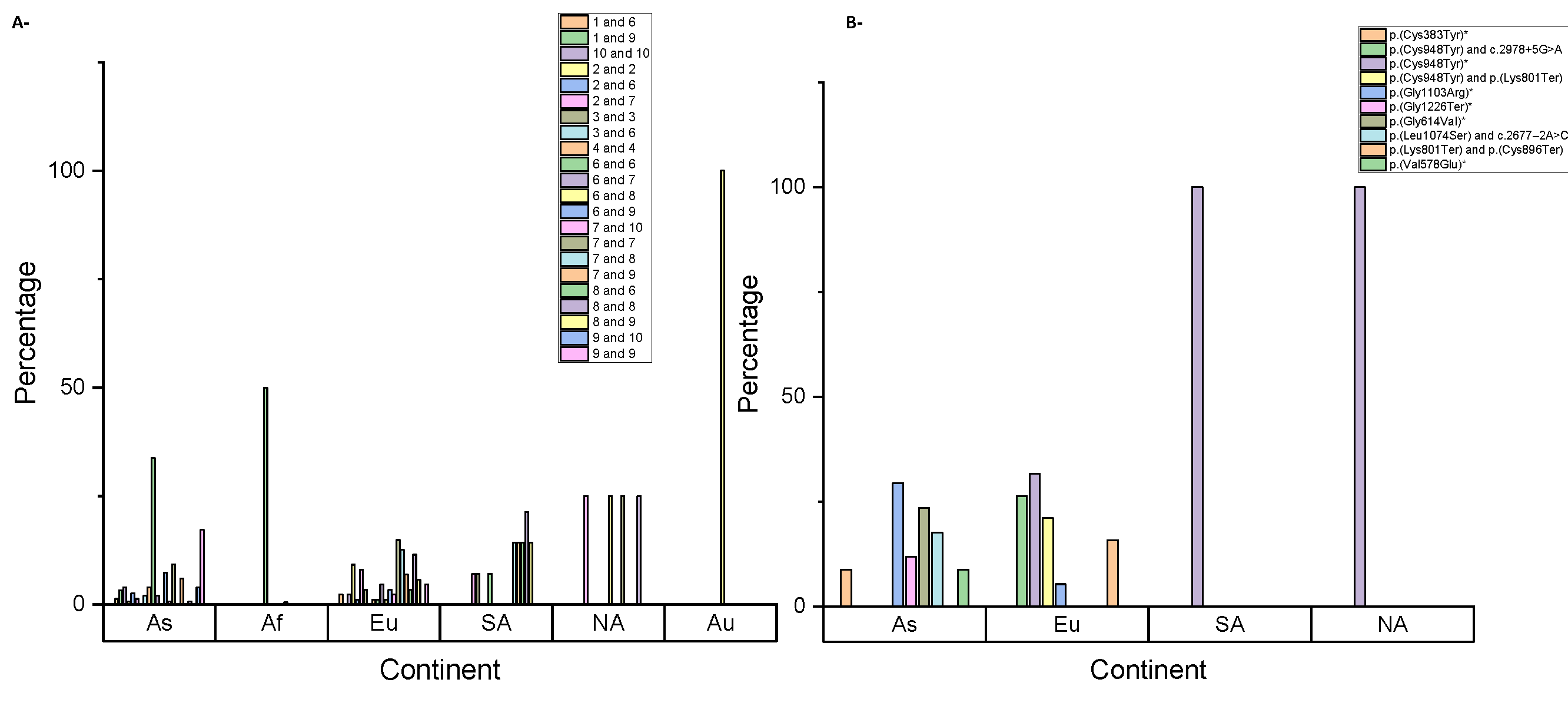


*Y axis: Percentage*

As: Asians, Af: Africans, Eu: Europeans, SA: South Americans, NA: North Americans, Au: Australia.
